# Step width variability as a discriminator of age-related gait changes

**DOI:** 10.1186/s12984-020-00671-9

**Published:** 2020-03-05

**Authors:** Andreas Skiadopoulos, Emily E. Moore, Harlan R. Sayles, Kendra K. Schmid, Nicholas Stergiou

**Affiliations:** 1 Department of Biomechanics and Center for Research in Human Movement Variability, University of Nebraska at Omaha, Biomechanics Research Building 214, 6160 University Drive South, Omaha, NE 68182-0860 USA; 2 Department of Health and Nutritional Sciences, South Dakota State University, Brookings, USA; 3 Department of Biostatistics, University of Nebraska Medical Center, Omaha, USA; 4 College of Public Health, University of Nebraska Medical Center, Omaha, USA

**Keywords:** Walking, Gait, Biomechanics, Rehabilitation, Lateral stability, Biomarkers

## Abstract

**Background:**

There is scientific evidence that older adults aged 65 and over walk with increased step width variability which has been associated with risk of falling. However, there are presently no threshold levels that define the optimal reference range of step width variability. Thus, the purpose of our study was to estimate the optimal reference range for identifying older adults with normative and excessive step width variability.

**Methods:**

We searched systematically the BMC, Cochrane Library, EBSCO, Frontiers, IEEE, PubMed, Scopus, SpringerLink, Web of Science, Wiley, and PROQUEST databases until September 2018, and included the studies that measured step width variability in both younger and older adults during walking at self-selected speed. Data were pooled in meta-analysis, and standardized mean differences (SMD) with 95% confidence intervals (CI) were calculated. A single-decision threshold method based on the Youden index, and a two-decision threshold method based on the uncertain interval method were used to identify the optimal threshold levels (PROSPERO registration: CRD42018107079).

**Results:**

Ten studies were retrieved (older adults = 304; younger adults = 219). Step width variability was higher in older than in younger adults (SMD = 1.15, 95% CI = 0.60; 1.70; *t* = 4.72, *p* = 0.001). The single-decision method set the threshold level for excessive step width variability at 2.14 cm. For the two-decision method, step width variability values above the upper threshold level of 2.50 cm were considered excessive, while step width variability values below the lower threshold level of 1.97 cm were considered within the optimal reference range.

**Conclusion:**

Step width variability is higher in older adults than in younger adults, with step width variability values above the upper threshold level of 2.50 cm to be considered as excessive. This information could potentially impact rehabilitation technology design for devices targeting lateral stability during walking.

## Background

Maintaining lateral stability during walking is a considerable challenge to the motor control system of the older adult, for whom age-related declines in sensorimotor functions could result in increased step width variability. Lateral stabilization during walking occurs due to the passive dynamics of the musculoskeletal system and the active control of the central nervous system [1, 2]. It has been also suggested that step width variability reflects the amount of active control that is required for lateral stabilization [3]. Accordingly, it appears that the age-related decrease in sensorimotor precision results in higher step width variability [4]. Based on this theoretical framework, when lateral foot placement becomes more stable the required amount of active control decreases, resulting in a consonant decrease in step width variability [1, 5].

Furthermore, evidence has surfaced to support the link between increased step width variability and risk of falling in older adults [6]. Step width variability was able to predict falls [7–10], and to differentiate older adults who fell from those who did not fall after a slip [11]. It could be suggested, therefore, that an intervention to decrease fall risk in older adults during walking could be effective if it targets to reduce the increased step width variability. However, a critical question for such an intervention to be successful is the amount of such a reduction. This is because the threshold level for identifying older adults with increased step width variability is presently unknown.

Thus, the purpose of this systematic review and meta-analysis was to establish the optimal threshold levels for identifying the boundaries of optimal reference range of step width variability. Such information could potentially impact the development of rehabilitation technology for devices targeting lateral stability to decrease risk of falling in older adults. In addition, it could potentially allow for better diagnostic and prognostic technology for individuals at risk of falling.

## Methods

### Protocol and registration

This systematic review and meta-analysis was conducted and reported in accordance with the preferred reporting items for systematic reviews and meta-analyses (PRISMA) statement, and the recommendations from the Cochrane collaboration initiative [12–15]. The review protocol and inclusion criteria were specified in advance based on the PRISMA-P statement and registered on the PROSPERO register of systematic reviews website (registration number: CRD42018107079). The PRISMA checklist is provided as Additional file 1.

### Literature review

A computerized systematic literature search, based on the Population Intervention Comparison Outcome (PICO) method was performed in the BMC, Cochrane Library, EBSCO, Frontiers, IEEE, PubMed, Scopus, SpringerLink, Web of Science, Wiley, and PROQUEST databases limited to research articles that have been published until September 2018. A string with Boolean search syntax operators was used to retrieve the titles and abstracts of the articles. The string contained all combinations of keywords and/or wildcards that specified the task, cohort, and outcome, combined with synonyms and terms from the MeSH thesaurus. The given query’s combinations were used to search the databases (see Additional file 2, which shows the search string).

### Inclusion criteria

Only peer-reviewed journal articles were included. Articles had to describe studies: (i) with samples whose participants were healthy younger (19–35 years) and older adults (65 years and over) free of overt neurological disorders and significant disabilities who were independently residing in the community; (ii) that measured step width during overground forward walking on a solid surface or on the treadmill at self-selected preferred speed, by using an optical system, pressure mat, instrumented walkway, or force plates; (iii) where all the participants had been measured under identical experimental conditions.

### Exclusion criteria

Articles were excluded if: (i) they were abstracts, conference proceedings, pilot studies, reviews with or without meta-analysis, qualitative studies, and technical reports; (ii) participants walked with an assistive device or had progressive neurological conditions, neurological impairments, lower limb disabilities, injuries or diseases that influence gait, or taking medications known to influence gait; (iii) participants walked over obstacles or sideways, backward, and not in a straight line or without a constant walking speed (e.g., accelerated, or decelerated walking speed); (iv) a metronome was used during walking (auditory, visual or any other sensorimotor stimulus or feedback); (v) the described studies used non-representative samples; (vi) they were published in languages other than English.

### Study selection

After removal of duplicate items, the titles and abstracts of the articles were screened independently by two review team members and excluded according to the predefined criteria; disagreement between reviewers was settled by consensus. Further, the reference list of each included article was checked and screened with the initial screening criteria to identify additional studies. This step was repeated until there were no further candidates for inclusion.

### Assessment of Methodologic quality

Quality has been assessed independently by two reviewers using an adaptation of the Downs and Black quality index checklist [16]. This scale is considered appropriate for assessing both randomized and non-randomized studies and provides an overall study quality score and score profiles for quality reporting, internal validity, power, and external validity [16]. Moreover, the method used by the authors to compute step width variability was evaluated. Only studies that computed step width variability as the standard deviation of the mediolateral distance between left and right foot during forward walking, whose coordinates were defined either on a global or a local reference system, and the anteroposterior axis of the reference system was matched with the direction of walking have been chosen. Disagreement between reviewers was settled by consensus.

### Data extraction

The data extracted by two reviewers were: age, sample size, exposure, preferred walking speed, step width variability (standard deviation of step width), step width calculation, and the instrumentation used for measuring step width. Measurements that reported stride width or base of support during walking were considered synonyms to step width since all measured mediolateral distance between feet. Variability in these measures was treated as equivalent to step width variability. The term lateral variability was treated as equivalent to step width variability, as well. When required, underlying numerical data were extracted through scaling of graphical representation. To reduce any error in this procedure, numerical data were extracted 10 times and mean values were computed and recorded as the measure of step width variability. When needed, the standard deviation of the step width variability was obtained from the parameters of the statistical analysis (*p*-value, *t*-value, standard error).

### Quantitative synthesis

Data were analyzed using standard meta-analytic modeling in R statistical 3.6.1 software with the meta, metafor, and dmetar packages [17–20]. Considerable heterogeneity was expected between the studies. Therefore, a meta-analysis of the mean difference in step width variability between older and younger adults’ groups was conducted using a random effects model, and Cohen’s method for pooling standardized mean differences (SMD) [21, 22]. Because of the small number of pooled studies and the expected heterogeneity the standard error estimates were adjusted using the Hartung-Knapp-Sidik-Jonkman correction [23–25] Confidence intervals for the SMD reported in each study and the overall SMD estimate were presented using forest plots.

Heterogeneity between studies was assessed using Cochran’s *Q* statistic [26]. Moreover, the *τ*^2^, *H*, and the Higgins’*I*^2^ measures for statistical heterogeneity were also incorporated as a cross-check [27, 28]. We performed influence and graphical display of study heterogeneity (GOSH) diagnostics, including leave-one-out analysis [29–32]. GOSH is a novel all-subsets combinatorial meta-analysis approach that calculates the effects sizes of 2^10^–1 subgroup to explore heterogeneity. Publication biases were evaluated visually with a contour-enhanced Funnel plot and formally checked by Begg’s and Egger’s tests [33–35].

### Identification of the optimal threshold levels of step width variability

The upper threshold level of step width variability set the bound of excessive step width variability. To identify the threshold level, a binary logistic regression analysis was conducted with aging (0 = younger adults; 1 = older adults) as the dependent variable, and the younger and older adults’ group averages of step width variability values across studies as the predictor variable. Therefore, the regression analysis was performed on group averages. It was assumed that the step width variability values in healthy younger adults set the optimal reference range. The Hosmer-Lemeshow chi-square test was used to measure model’s goodness of fit [36]. Non-significant chi-square indicates a failure to reject the null hypothesis implying that the model’s estimates fit the data at an acceptable level. The receiver-operating characteristic (ROC) curve was used to evaluate the discrimination ability of the binary logistic model by calculating the area under the ROC curve (AUC). The cutoff value to identify the excessive step width variability was estimated from the ROC analysis using Youden’s index.

We expected that there would be a degree of overlap between the step width variability values of the younger and older adults’ groups. Thus, a novel trichotomization method was used also to provide two threshold levels that define an interval of uncertainty around Youden index. Around Youden index the step width variability values are inter-mixed and have a near equal probability of indicating ‘reference’ or ‘excessive’ providing little or no information whether an individual is a younger or older adult [37]. The cutoff points of the uncertain interval method were chosen at specificity (Sp) = sensitivity (Se) = 0.50.

## Results

### Study selection

**Fig. 1:**
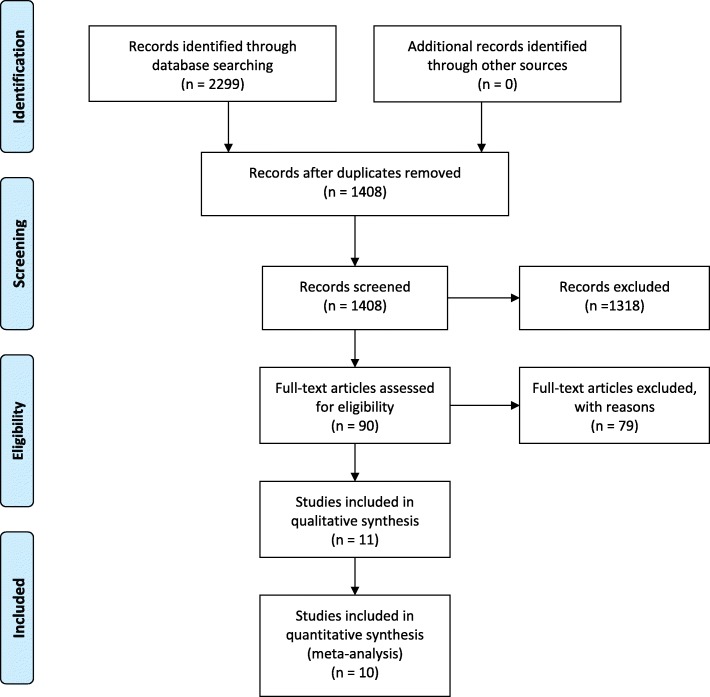
Study selection flowchart

Figure 1 displays a flowchart summarizing the results of the literature search. In summary, from the 1408 unduplicated studies identified, 1318 of them were excluded during the title and abstract screening, and from the remaining 90 full-text reviewed articles, 79 of them were excluded after full-text screening because step width variability values were not stated, subjects did not walk at preferred speed, the age of the older adults group was not over 65 years, did not include both older and younger groups or because the pace of the preferred speed was maintained by an auditory metronome. The remaining 11 articles were included in the qualitative synthesis [38–48] (see Additional file 3, for detailed flow diagram). Of the 11 eligible studies, two studies recruited the same younger adults’ population [39, 40]. In one study the same younger and older adults’ populations contributed twice; in a repeated single and in a continuous overground walking [46]. The continuous protocol has been chosen because it involves walking without interruptions [46].

### Study subjects

Table 1 reports the characteristics of the 11 studies included in the systematic review. All studies used slightly different cut points to distinguish younger from old. Of the 11 studies, two studies [46, 47] recruited only female subjects; one study [45] only reported the total (male and female) subjects. Data were extracted from 323 older adults with mean age 74.41 ± 6.29 years old, and 239 younger adults with mean age 25.3 ± 4.6 years old. Eight studies assessed health of the younger or older adults’ population by medical history and/or physical, psychiatric or neurological examinations [38–41, 43, 44, 46, 48]. One assessed only the health of the older population [45], and one study detailed specific exclusion criteria [47]. One study did not specify any physical or other examinations for assessing subjects’ health [42].

**Table 1:** Study summary for the step width variability during walking in young and older adults

Author / Year	Sample size	Exposure	Preferred walking speed protocol	Step width calculation	Step width variability / analyzed steps (avg.)
Almarwani et al. (2016a) [39]	OA, *n* = 111 (82 f), age = 77.25 ± 6.0 yr., ht. = 163.4 ± 9.5 cm, wt. = 77.4 ± 15.7 kg; YA, *n* = 40 (30 f), age = 26.60 ± 6.0 yr., ht. = 168.4 ± 8.3 cm, wt. = 66.4 ± 12.4 kg	Walking on a 4 m walkway in 3-speed conditions	Participants were instructed to walk at a pace that represented their usual walking speed. OA, PWS = 1.07 ± 0.26 m/s; YA, PWS = 1.29 ± 0.19 m/s.	The distance between the outermost borders of two consecutive footprints (GaitMat II).	OA: 3.70 ± 1.80 cm; YA: 2.40 ± 0.60 cm. OA: 23 steps YA: 38 steps
Almarwani et al. (2016b) [40]	OA, *n* = 46 (35 f), age = 78.09 ± 6.2 yr., ht. = *nr* cm, mass = *nr* kg; YA, n = 40 (30 f), age = 26.6 ± 6.0 yr., ht. = *nr* cm, mass = *nr* kg;	Walking on an 8 m walkway at preferred speed	Authors did not describe how the preferred walking speed was determined. OA, PWS = 0.95 ± 0.28 m/s; YA, PWS = 1.29 ± 0.19 m/s.	The distance between the outermost borders of two consecutive footprints (GaitMat II).	OA: 3.00 ± 1.41 cm; YA: 2.50 ± 1.41 cm OA: 23 steps YA: 38 steps
Decker et al. (2016) [41]	OA, *n* = 19 (9 f), age = 69.26 ± 1.11 yr., ht. = 171 ± 2 cm, mass = 77.45 ± 2.78 kg; YA, *n* = 20 (12 f), age = 24.45 ± 0.87 yr., ht. = 173 ± 2 cm, mass = 70.41 ± 2.63 kg	Three-minutes treadmill walking at 4 attentional demands conditions at a preferred speed	Participants started walking at a slow speed while the treadmill was slowly accelerated by 0.1 km/h until the participants reported their PWS. Then the speed was increased by 1.5 km/h and was slowly decreased by 0.1 km/h until the participants reported their PWS. This procedure was repeated until a less than 0.4 km/h difference was achieved. OA, PWS = 0.77 ± 0.04 m/s; YA, PWS = 1.06 ± 0.03 m/s.	Mediolateral distance between foot midpoints calculated over the consecutive instants when the left (or right) swing limb’s knee passed in front of the right (or left) stance limb’s knee	OA: 1.70 ± 0.17 cm; YA: 1.92 ± 0.08 cm OA: ≥ 256 steps YA: ≥ 256 steps
Ihlen et al. (2012) [42]	OA, *n* = 10 (4 f), age = 75.4 ± 4.6 yr., ht. = 170.9 ± 11.8 cm, mass = 76 ± 13.1 kg; YA, *n* = 10 (4 f), age = 25.7 ± 4.7 yr., ht. = 177.6 ± 8.3 cm, mass = 74.5 ± 9.5 kg	Ten-minutes treadmill walking in 3-speed conditions	Authors did not describe how the preferred walking speed was determined. OA, PWS = 1.17 ± 0.10 m/s; YA, PWS = 1.11 ± 0.15 m/s.	Step width was defined as the mediolateral distance between heel markers at the time of heel strike	OA: 2.55 ± 0.35 cm; YA: 1.91 ± 0.30 cm OA: *nr* steps YA: *nr* steps
Kang et al. (2008) [43]	OA, n = 18 (6 f), age = 72.1 ± 6.0 yr., ht. =170 ± 10.4 cm, mass = 73.2 ± 12.3 kg; YA, *n* = 17 (5 f), age = 23.6 ± 2.6 yr., ht. = 173 ± 9.4 cm, mass = 71.1 ± 9.86 kg	Five-minutes treadmill walking in 5-speed conditions	Participants reported the limits of their preferred speed while the treadmill was slowly accelerated, then decelerated three times. These upper and lower limits were averaged to determine their preferred walking speed. OA, PWS = 1.29 ± 0.15 m/s; YA, PWS = 1.30 ± 0.10 m/s.	Step width was defined as the distance between the heel and the contralateral heel at each heel contact in the mediolateral direction	OA: 2.14 ± 0.54 cm; YA: 2.01 ± 0.56 cm OA: *nr* steps YA: *nr* steps
Lovden et al. (2008) [38]	OA, n = 32 (16 f), age = 73.6 ± 2.9 yr., ht. = 169.4 ± 10 cm, mass = 74.3 ± 11.5 kg; YA, *n* = 32 (16 f), age = 25.0 ± 2.9 yr., ht. = 177.6 ± 9.8 cm, mass = 71.6 ± 13.1 kg	Twenty-secs treadmill walking in 4 conditions of working memory load at a preferred speed	Participants gradually increased speed to determine preferred walking speed. After walking at their self-selected speed for 3 min were asked again if they felt comfortable with their choice. OA, PWS = 0.87 ± 0.13 m/s; YA, PWS = 1.04 ± 0.11 m/s.	The step width was measured as the perpendicular distance between the line of progression and the heel location of the contralateral foot.	OA: 2.19 ± 0.11 cm; YA: 1.97 ± 0.12 cm OA: 18 steps YA: 18 steps
Marigold et al. (2008) [44]	OA, n = 10 (5 f), age = 74.1 ± 7.2 yr., ht. = *nr* cm, mass = *nr* kg; YA, n = 10 (5 f), age = 26.1 ± 5.2 yr., ht. = *nr* cm, mass = *nr* kg	walking on a multi-surface terrain in 4 different terrain configurations for YA and in 3 different conditions for OA, respectively, at a preferred speed	Authors did not describe how the preferred walking speed was determined. OA, PWS = 1.20 ± 0.12 m/s; YA, PWS = 1.32 ± 0.16 m/s.	The mediolateral distance between ankle markers	OA: 4.09 ± 0.70 cm; YA: 2.96 ± 1.29 cm OA: *nr* steps YA: *nr* steps
Owings et al. (2004a) [45]	OA, *n* = 12 (nr), age = 73.4 ± 2.3 yr., ht. = 172 ± 13 cm, mass = 76.3 ± 15.5 kg; YA, *n* = 18 (nr), age = 27.7 ± 3.3 yr., ht. = 168 ± 11 cm, mass = 35.9 ± 10.2 kg	Ten-minutes treadmill walking for OA and 15-min for YA at a preferred speed	Authors did not describe how the preferred walking speed was determined. OA, PWS = 0.97 ± 0.17 m/s; YA, PWS = 1.06 ± 0.28 m/s.	Step width was determined as the mediolateral distance between the sequential left and right heel-strikes	OA: 2.50 ± 0.70 cm; YA: 2.10 ± 0.50 cm OA: *nr* steps YA: *nr* steps
Paterson et al. (2009) [46]	OA, n = 32 (32 f), age = 67.4 ± 6.3 yr., ht. = 162 ± 7 cm, mass = 65.1 ± 13.2 kg; YA, *n* = 22 (22 f), age = 21.2 ± 2.5 yr., ht. = 166 ± 8 cm, mass = 62.6 ± 9.8 kg	10 m continuous laps of a walking circuit at a preferred speed	Authors did not describe how the preferred walking speed was determined. OA, PWS = nr; YA, PWS = nr.	The midline midpoint of the current footprint to the midline midpoint of the previous footprint on the opposite foot (GaitRite).	OA: 2.50 ± 0.83 cm; YA: 1.90 ± 0.83 cm OA: *nr* steps YA: *nr* steps
Thies et al. (2005) [47]	OA, n = 12 (12 f), age = 70.2 ± 4.1 yr., ht. = *nr* cm, mass = *nr* kg; YA, n = 12 (12 f), age = 22.2 ± 3.0 yr., ht. = *nr* cm, mass = *nr* kg,	Walking on a 10 m walkway in 4 task conditions at a preferred speed	Subjects were asked throughout the experiment to walk at a comfortable speed as if they were going to mail a letter. OA, PWS = 1.15 ± 0.06 m/s; YA, PWS = 1.08 ± 0.06 m/s.	Mediolateral distance between the left and right foot ankle (tibiotalar joint) markers during double support	OA: 2.99 ± 0.20 cm; YA: 2.50 ± 0.17 cm OA: 55 steps YA: 63 steps
Woledge et al. (2005) [48]	OA, *n* = 21 (8 f), age = 72.7 ± 1.21 yr., ht. = 166 ± 2 cm, mass = 68.3 ± 2.6 kg; YA, n = 17 (11 f), age = 27.3 ± 1.5 yr., ht. = 171 ± 2 cm, mass = 64.3 ± 2.9 kg	Walking on 8 m walkway at a preferred speed	Authors did not describe how the preferred walking speed was determined. OA, PWS = 1.12 ± 0.06 m/s; YA, PWS = 1.19 ± 0.03 m/s.	The lateral difference between successive footfall positions (medial malleoli)	OA: 2.32 ± 0.28 cm; YA: 1.73 ± 0.37 cm OA: 34 steps YA: 34 steps

*Note. OA* older adults; *YA* younger adults; *PWS* preferred walking speed; *f* females; *nr* not reported; *n* number; *yr* years; *m/s* meters per second; *cm* centimeters; *avg* average; *ht* height

### Instrumentation used for measuring step width

Six studies measured step width during overground walking [39, 40, 44, 46–48] and five studies during walking on a treadmill [38, 41–43, 45]. Of the six overground walking studies, five studies [39, 40, 44, 47, 48] used repeated single walking protocols and one study [46] both repeated single and continuous walking protocols. Data collection during overground walking was done by using either an instrumented walkway [39, 40, 46] or a motion capture system [44, 47, 48]. During treadmill walking data were collected using motion capture systems [38, 41–43] or force plates (i.e., center of pressure - COP) [45]. In addition, Table 2 reports the quality assessment performed on the selected studies used in the meta-analysis.

**Table 2:** Methodologic assessment of study design quality using an adaptation of the quality index [16]. Numbering refers to the quality index item

ITEM	STUDY										
	Almarwani et al. (2016a) [39]	Almarwani et al. (2016b) [40]	Decker et al. (2016) [41]	Ihlen et al. (2012) [42]	Kang et al. (2008) [43]	Lovden et al. (2008) [38]	Marigold et al. (2008) [44]	Owings et al. (2004a) [45]	Paterson et al. (2009) [46]	Thies et al. (2005) [47]	Woledge et al. (2005) [48]
1. Is the hypothesis/aim/objective of the study clearly described?	1	1	1	1	1	1	1	1	1	1	1
2. Are the main outcomes to be measured clearly described in the Introduction or Methods section?	1	1	1	1	1	1	1	1	1	1	1
3. Are the characteristics of the participants included in the study clearly described?	1	1	1	0	1	1	1	0	0	1	1
5. Are the distributions of the principal confounders in each group of subjects to be compared clearly described?	2	2	2	2	2	2	2	2	2	2	2
6. Are the main findings of the study clearly described?	1	1	1	1	1	1	1	1	1	1	1
7. Does the study provide estimates of the random variability in the data for the main outcomes?	1	1	1	1	1	1	1	1	1	1	1
10. Have actual probability values been reported (e.g. 0.035 rather than < 0.05) for the main outcomes except where the probability value is less than 0.001?	1	1	1	0	1	0	1	1	1	1	1
11. Were the subjects asked to participate in the study representative of the entire population from which they were recruited?	1	1	0	0	1	1	1	0	0	1	1
12. Were those subjects who were prepared to participate representative of the entire population from which they were recruited?	0	0	0	0	0	0	0	0	0	0	0
13. Were the staff, place and facilities where the study was set representative of a laboratory environment?	1	1	1	1	1	1	1	1	1	1	1
16. If any of the results of the study were based on “data dredging”, was this made clear?	1	1	1	1	1	1	1	1	1	1	1
20. Were the main outcome measures used accurate (valid and reliable)?	1	1	1	1	1	1	1	1	1	1	1
21. Were the subjects recruited from the same population?	1	1	0	0	0	1	1	0	0	1	0
22. Were study subjects recruited over a defined period of time?	0	0	0	0	0	0	0	0	0	0	0
25. Was there adequate adjustment for confounding in the analysis from which the main findings were drawn?	1	1	1	1	1	1	1	1	1	1	1
27. Did the study have a power analysis?	0	0	0	0	0	0	0	0	0	0	0
TOTAL	14	14	12	10	13	13	14	11	11	14	13

### Assessment of publication Bias and heterogeneity sensitivity analysis

**Fig. 2:**
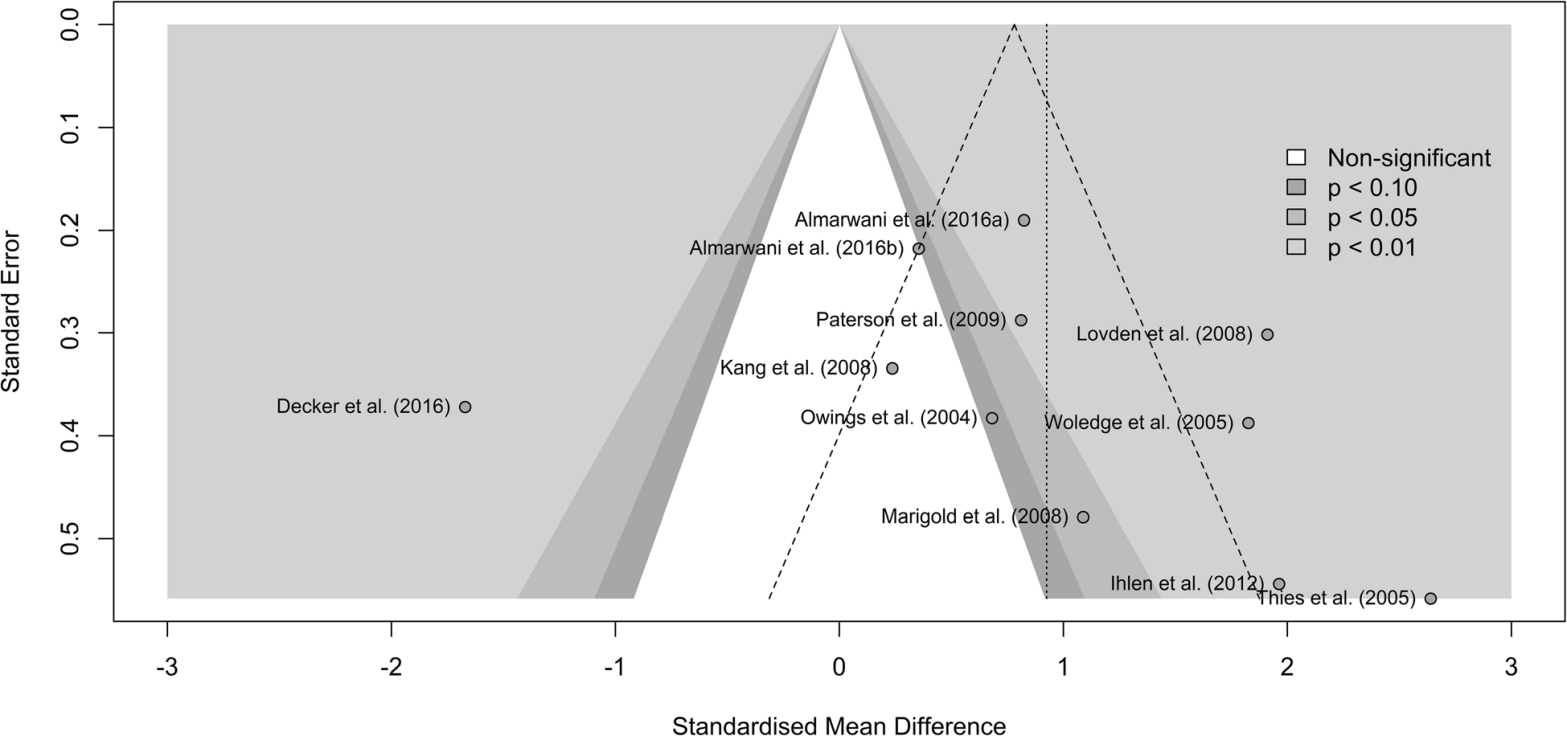
Contour-enhanced Funnel plot of standardized mean differences. Standardized mean differences in the white area are statistically non-significant (*p* > 0.1). The dashed angled lines represent the bounds within which 95% of studies should fall if there is no statistical heterogeneity. The dashed vertical line represents the estimate for the overall effect from the random-effect model

Visual inspection of the contour-enhanced funnel plot (Fig. 2) indicated the presence of publication bias. Neither Begg’s rank correlation test (*z* = 1.79, *p*-value = 0.07) nor Egger’s regression test (*t* = 1.99, *df* = 8, *p*-value = 0.08) returned statistically significant results. Diagnostics plots identified the study of Decker et al., [41] as a potential outlier (see Additional file 4). Thus, we omitted the study of Decker et al., [41] from the meta-analysis. Therefore, for the meta-analysis, data were extracted from the 10 remaining studies.

### Step width variability in younger adults vs older adults

**Fig. 3:**
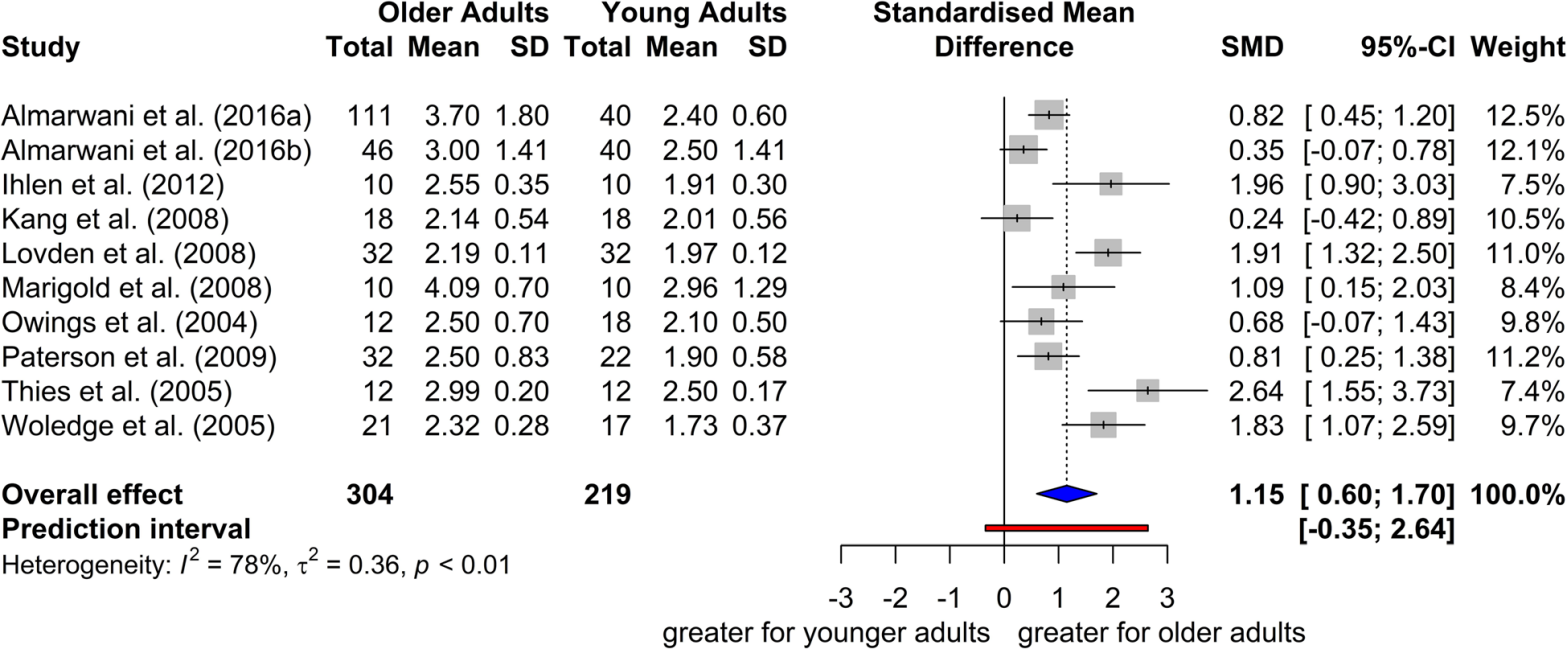
Forest plot of standardized mean difference (SMD) and 95% confidence intervals (CI) for the step width variability between older and younger adults. The difference found for the step width variability between the younger and older adults was statistically significant (*p* = 0.001) indicating that step width variability was higher in older adults than in younger adults. SD: standard deviation, SMD: standardized mean difference, CI: confidence interval

The study of Decker et al., [41] was omitted from the meta-analysis as an influential outlier, and the meta-analysis was conducted with the 10 remaining studies (304 older adults with mean age 74.74 ± 6.34 years old, and 219 younger adults with mean age 25.4 ± 4.8 years old). The meta-analysis revealed a significant overall effect size (SMD = 1.15, 95% CI = 0.60; 1.70; *t* = 4.72, *p* = 0.001), indicating that step width variability was higher in older adults than in younger adults (Fig. 3). The between studies heterogeneity was moderate (*τ*^2^ = 0.36, H = 2.14 [1.58; 2.89]; *I*^2^ = 78% [60%; 80%]; *Q* = 41.14, *p*-value < 0.001). Two of the studies recruited only female participants [46, 47]. Subgroup meta-analysis using a mixed-effects model (random-effects model within subgroups, fixed-effects model between subgroups) [17] was conducted to test whether gender modified the meta-analytic results. The test for subgroup differences indicated that there is no statistically significant subgroup effect (*p* = 0.52) (analysis not presented). Additionally, a subgroup meta-analysis was conducted to test if heterogeneity varies according to walking environment (‘overground walking’ vs. ‘treadmill walking’). The test for subgroup differences indicated that there is no statistically significant subgroup effect (*p* = 0.94).

### Discrimination of step width variability for predicting age group

The binary logistic regression showed a good association (*z* = − 1.928, *p* = 0.057) and a good accuracy (ACC) (correct classification of older and younger adults’ group) for the step width variability to predict subjects’ age group (younger adults vs. older adults) (ACC = 0.70; no information rate (NIR) = 0.50; *p*-value [ACC > NIR] = 0.06; Hosmer-Lemeshow: *χ*^2^ = 7.58, *p* = 0.37). Using the ROC curve approach, the Youden’s index value was chosen as the cutoff value, which corresponds to step width variability value of 2.14 cm. The accuracy of the cutoff value based on the dichotomization approach (Youden’s index) was ACC_di_ = 0.80 of the older and younger adults’ groups. The sensitivity (older adults’ groups with excessive step width variability) was Se_di_ = 1.00, and the specificity (younger adults’ groups with healthy step width variability) was Sp_di_ = 0.60. The positive predictive value (probability to belong to the older adults’ groups when step width variability is excessive) was PPV_di_ = 0.71, and the negative predictive value (probability to belong to the younger adults’ groups when step width variability is healthy) was NPV_di_ = 1.00.

**Fig. 4:**
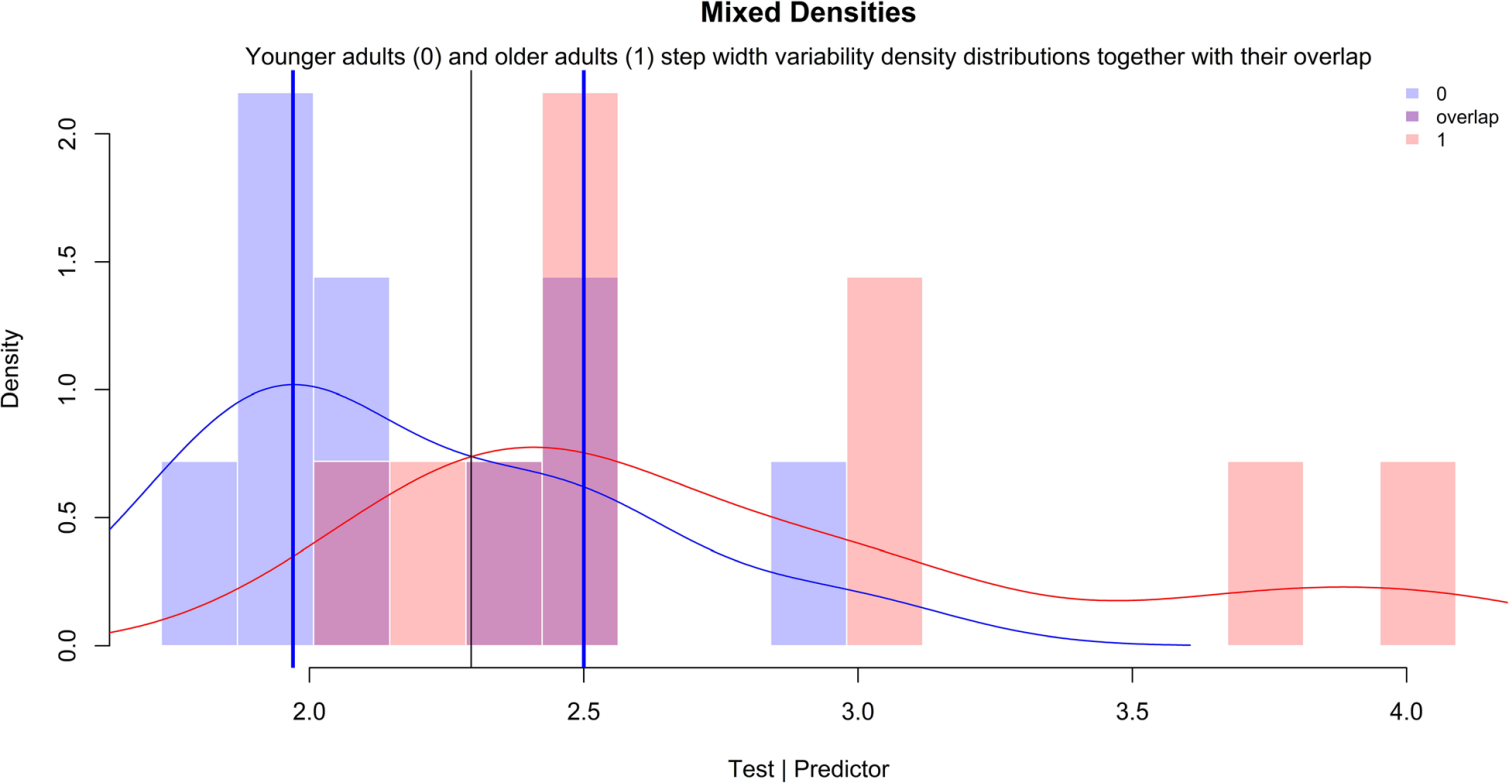
Visual inspection of the uncertain interval method. The densities of older adults and younger adults step width variability distributions together with their overlap are presented. Youden index occurs at the intersection of both density distributions, where the overlap is higher (0 = younger adults; 1 = older adults). The blue vertical lines are the optimal threshold levels

Using the approach of Landsheer, [37] the optimal reference range was separated from the excessive step width variability by an uncertainty interval. The lower and upper threshold levels of the uncertainty interval were Lo = 1.97 cm and Hi = 2.50 cm, respectively. Eleven observations were considered as uncertain (Table 3). The uncertain step width variability of the younger adults was compared with that of the older adults. The *t*-test did not reveal statistically difference between the two groups (*t* = − 0.13, *p* = 0.89). The trichotomization approach improved accuracy (ACC_tr_ = 0.88) (Table 3). Moreover, the sensitivity (Se_tr_ = 1.00), specificity (Sp_tr_ = 0.75), positive predicted value (PPV_tr_ = 0.83), and negative predicted value (NPV_tr_ = 1.00) were improved. Within the interval it is impossible to decide about the absence or not of excessive step width variability (CCR_un_ = 0.55; Se_un_ = 0.60; Sp = 0.50; PPV_un_ = 0.50; NPV_un_ = 0.60). The trichotomization approach removed inter-mixed step width variability values providing more information for the classification. Therefore, step width variability values above the threshold level of Hi = 2.50 cm were considered excessive, while step width variability values below the threshold level of Lo = 1.97 cm were considered within the optimal reference range (Fig. 4).

**Table 3:** Confusion matrix for the uncertain interval method

Classified	Actual	
	Younger adults	Older adults
Younger adults (SWV < Lo)	3	0
Uncertain Interval (Lo ≤ SWV ≤ Hi)	6	5
Older adults (SWV > Hi)	1	5

*Notes: SWV* step width variability; *Lo* 1.97 cm; *Hi* 2.50 cm

## Discussion

### Implications for clinical practice

In this systematic review and meta-analysis we sought to define the optimal threshold levels for identifying the boundaries of optimal reference range of step width variability in older adults. As such, we provided evidence of optimal threshold levels of step width variability with an uncertainty interval. For our purposes, the step width variability values in healthy younger adults set the optimal reference range.

Lateral foot placement has been shown to be the dominant mechanism that ensures lateral stability during walking [49, 50]. Simple locomotion models suggest that lateral stability is controlled through active adjustments of lateral foot placement which is determined from integrative sensory feedback with each step [1, 3, 51, 52]. Based on this approach, step width variability serves as an indicator of the required active control, [3] and as a quantifier of the degree to which sensory inputs contributes to the active control [53, 54]. As such, step width variability increases when active control is subjected to noisy inputs [5]. Age-related decrease in sensorimotor precision [55–57] can be treated as a reduced signal-to-noise ratio [5, 58, 59]. It is likely that an imprecise active control in older adults *causes* increments in step width variability [5, 60] and increases the risk of falling [52]. Indirect evidence comes from many studies demonstrating an increase in step width variability with aging [45, 48, 61, 62] but not between populations of older adults with different balance control abilities [63]. The clinical utility for identifying the older adults with excessive step width variability relies on previous research work that has related increased step width variability in older adults with increased risk of falling [11, 64]. Mechanically, since foot placement is the dominant mechanism that ensures lateral stability during walking, [2, 49, 50] the alignment of the step width variability to the optimal reference values would reflect an improvement in the lateral stability. While age-related, non-pathological decline in walking performance occurs in everybody, older adults who walk with excessively increased step width variability are at a higher risk of falling [11]. Therefore, it is plausible that older adults at decreased risk of falling walk within the optimal reference range of step width variability, while older adults at high risk of falling walk with excessive, non-optimal reference range values. The comparison with reference values could be set realistic goals for interventions targeted to improve lateral stability during walking. The proposed reference values can be used to express older adults’ step width variability as a percentage of what individuals with precise active control can achieve.

Gait training has become an essential component of fall prevention interventions and is recommended in current fall prevention guidelines [65]. The extent to which a gait training intervention has the potential to ameliorate the common age-related deterioration in gait performance of older adults and reduce risk of falling is dependent on the specific population being examined. It can be argued that gait training requires a measure of gait performance that can be used both to *profile* older adults for screening practices, and as a sensitive indicator for *monitoring* an individual’s performance. Our results suggest that gait training would be more effective in decreasing risk of falling in older adults if it targets to align excessive step width variability to the optimal reference range values. For example, Wang et al., [66] proposed a 12-week exercise intervention able to decrease gait variability in older adults. However, it is impossible to know whether postintervention gait variability fell within normal values in the absence of an optimal reference range. Step width variability could be used to identify older adults at higher risk of falling, and as a biomarker to be targeted for gait training intervention. Step width variability is a straightforward measurement due to its simplicity, it is noninvasive, easy to perform, and inexpensive. Such information could be implemented in the development of rehabilitation technology for devices targeting lateral stability to decrease risk of falling in older adults.

Recently, it has been shown that a change in attentional demands causes a consonant decrease or increase in step width variability in older adults during treadmill walking (a U-shaped relation) [41]. In this study, step width variability in the most cognitively demanding condition did not exceed that of the control walking condition (i.e., without any attentional demands). This was interpreted as a protective mechanism of the central nervous system to counteract the increased risk of falling that is related with excessive step width variability. However, in the absence of an optimal reference range, we do not know who walk within, near or below to the boundary of excessive step width variability. This meta-analysis contributes to fill this essential gap of knowledge. As the effectiveness of any intervention is related to the specific population being examined, using optimal threshold levels for step width variability can allow the selection of older adults with excessive (or normal) step width variability in the absence of attentional demands other than that of the walking activity itself.

### Computation of step width variability

High and low step width variability values (low, < 7–8%; moderate, 8–27%; high, > 27–30%), expressed as the coefficient of variation of the step width, has been related retrospectively with falls and with low levels of physical activity in older adults who did not walk slowly (i.e., gait speed ≥1 m/s) [8, 67]. The coefficient of variation of step width variability has been questioned as being an appropriate parameter to express step width variability during walking. Helbostad and Moe-Nilssen, [68] showed that the coefficient of variation of the step width demonstrates ‘a spurious relation to gait speed’ because the mean step width value is non-linearly associated with walking speed (a U-shaped relation). On the other hand, the standard deviation of step width demonstrates no relation with walking speed indicating that the within-subject standard deviation of step width could be a more suitable parameter to express step width variability [43, 68]. In addition, the coefficient of variation depends on the foot markers used to calculate the step width. Woledge et al., [48] defined step width as the mediolateral distance between the left and right medial malleoli during double support, while Owings and Grabiner, [45] defined it as the mediolateral distance between the sequential left and right heel markers. In other words, if we had collected data on the same subject during walking, the use of different foot markers to calculate step width would have resulted in different coefficient of variations, while the standard deviations of the step width would have been the same (assuming that the foot is a rigid segment and there is no rotation). This is supported by a recent literature review that showed that the coefficient of variation of step width exhibited large differences between studies [69]. Finally, the coefficient of variation is applicable only to ratio data, and the step width is considered interval data as it is not bounded by a meaningful zero point [46, 70]. Therefore, in this systematic review and meta-analysis we included only studies that reported the step width variability as the standard deviation of the step width.

### Strength and limitations of the analysis

We provided scientific evidence to use step width variability as an age determinant of gait control. All participants in the studies identified were considered healthy younger and older adults free of overt neurological disorders and significant disabilities who were independently residing in the community. However, subclinical gait deficiencies that occur with aging could result in increased step width variability. In our study we identified boundaries of optimal reference range of step width variability, and we provide the highest level of evidence that step width variability in older adults is higher than that of younger adults. However, our study cannot claim that this optimal reference range of step width variability can discriminate fallers and non-fallers in older adults. Future research should investigate this question and endeavor to investigate whether fall risk among older adults could be reduced by decreasing the excessive step width variability. This would set step width variability as a robust and sensitive marker to be targeted for intervention to ameliorate age-related deterioration in lateral stability during walking.

However, our results should be considered in lieu of certain limitations. A key requirement for maximizing the likelihood to detect a true difference between younger and older adults is to perform a power analysis beforehand. Any lack of accuracy or reliability of the step width variability measurement can reduce the likelihood of detecting a true difference (study power). For example, it has been suggested that for treadmill walking, an accurate measure of step width variability can be achieved with at least 400 steps (i.e., about 10 min treadmill walking) [71, 72]. Of the seven included studies that used treadmill, only three had a 10 min walking protocol. Similarly, it has been showed that reliability (minimum detectable change) of step width variability during overground walking improves with an increase in sample size (i.e., number of steps) [39, 73]. Thus, longer evaluations of step width variability during overground and treadmill walking are necessary to obtain accurate and reliable measurement of step width variability. Nevertheless, this may impose an unnecessary burden for older adults due to physical limitations and it can introduce confounding factors like fatigue [74]. Another drawback is the difficulty to measure step width variability on overground walking over long straight distances. To overcome this drawback repeated trials of consecutive steps can be measured during overground walking. Repeated short duration measurements of continuous overground walking protocols are preferred because are more reliable than repeated single walking protocols [46]. Of the six overground studies, five studies used repeated single walking protocols and only one study continuous walking protocol (Table 2). Regardless of such limitations, we still support that a meta-analysis is the best level of evidence providing the least-biased estimate. An additional limitation of our meta-analysis is that the probabilistic approach we used to estimate threshold levels of step width variability, was based on group data rather than on individual data. Further research to explore any loss in information in our meta-analytic approach is necessary.

## Conclusions

In summary, older adults walk with higher step width variability than younger adults. Older adults who walk with step width variability values above the upper threshold level of 2.50 cm, could be characterized as having excessive step width variability. This information could potentially impact rehabilitation technology design for devices targeting lateral stability during walking.

## Supplementary information

**Additional file 1.** PRISMA Checklist.

**Additional file 2.** Search strategy.

**Additional file 3.** Detailed flow diagram of literature search and selection of studies.

**Additional file 4.** Heterogeneity analyses.

## Data Availability

The dataset supporting the conclusions of this article is included within the article (and its additional file).

## Abbreviations

ACC: Accuracy
AUC: Area under curve
CCR: Cutoff correctly classified
GOSH: Graphical display of study heterogeneity
Hi: High
Lo: Low
NIR: No information rate
NPV: Negative predictive value
PICO: Population Intervention Comparison Outcome
PPV: Positive predictive value
PRISMA: Preferred Reporting Items for Systematic Reviews and Meta-analyses
ROC: Receiver-operating characteristic
Se: Sensitivity
SMD: Standardized mean differences
Sp: Specificity
SWV: Step width variability
