# Supplementary material for: Step width variability as a discriminator of age-related gait changes
Source: J Neuroeng Rehabil. 2020 Mar 5;17:41. doi: 10.1186/s12984-020-00671-9 (PMC7059259; doi:10.1186/s12984-020-00671-9)
Supplement: Supplementary file 2 — Additional file 2. Search strategy. [file 12984_2020_671_MOESM2_ESM.pdf]

Additional Table 1. Full search string. PubMed database from inception to September 2018. The next steps were followed for the systematic literature review: i) Found MeSH words in PubMed; ii) Created query in PubMed; iii) Used given query's combinations to search databases (see Figure, Supplemental Figure S1).

Query
((((("Gait"[Mesh] OR "Walking"[Mesh]) AND "Aged"[Mesh]) OR "Aged, 80 and over"[Mesh]) OR "Healthy Aging"[Mesh] AND "Young Adult"[Mesh]) OR "Adult"[Mesh]) AND step[All Fields] AND width[All Fields] AND variability[All Fields]
