# Supplementary figures and images for: Step width variability as a discriminator of age-related gait changes
Source: J Neuroeng Rehabil. 2020 Mar 5;17:41. doi: 10.1186/s12984-020-00671-9 (PMC7059259; doi:10.1186/s12984-020-00671-9)

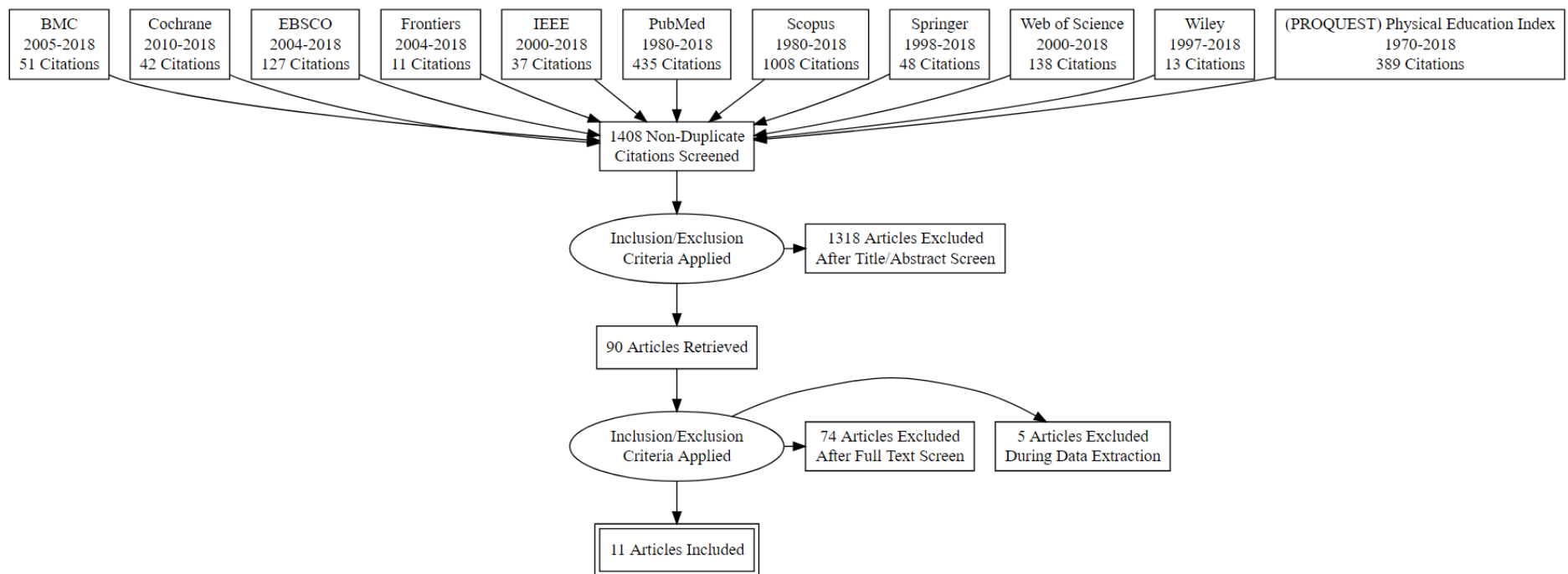

**Additional Figure 1:** Detailed flow diagram of literature search and selection of studies

Supplement: Supplementary file 3 — Additional file 3. Detailed flow diagram of literature search and selection of studies. [file 12984_2020_671_MOESM3_ESM.pdf]
