# Supplementary material for: Step width variability as a discriminator of age-related gait changes
Source: J Neuroeng Rehabil. 2020 Mar 5;17:41. doi: 10.1186/s12984-020-00671-9 (PMC7059259; doi:10.1186/s12984-020-00671-9)
Supplement: Supplementary file 4 — Additional file 4. Heterogeneity analyses. [file 12984_2020_671_MOESM4_ESM.pdf]

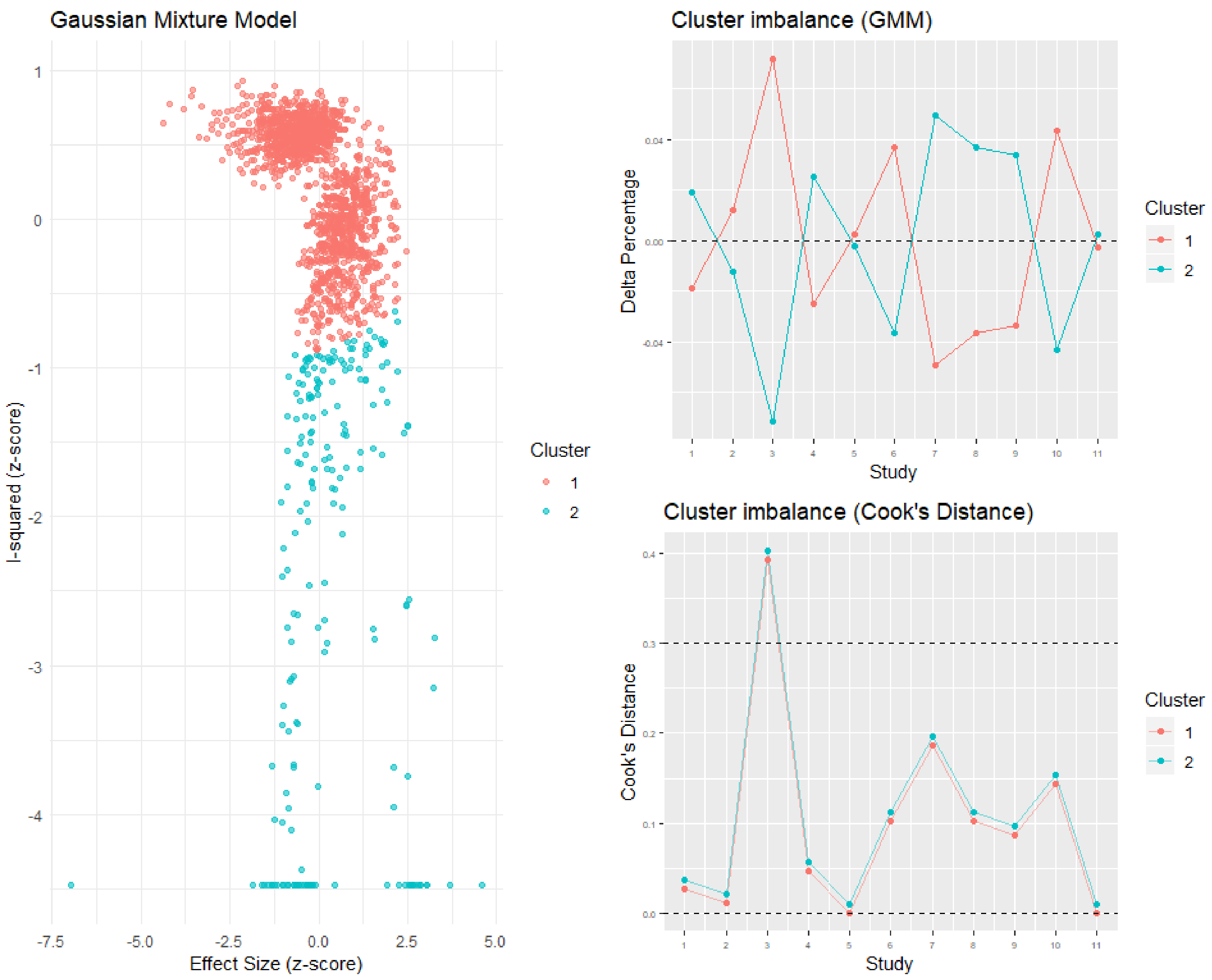

Gaussian Mixture Model
I-squared (z-score)
Effect Size (z-score)
1
0
-1
-2
-3
-4
-7.5
-5.0
-2.5
0.0
2.5
5.0
Cluster
1
2
Cluster imbalance (GMM)
Delta Percentage
0.04
0.00
-0.04
Study
1
2
3
4
5
6
7
8
9
10
11
Cluster imbalance (Cook's Distance)
Cook's Distance
0.4
0.3
0.2
0.1
0.0

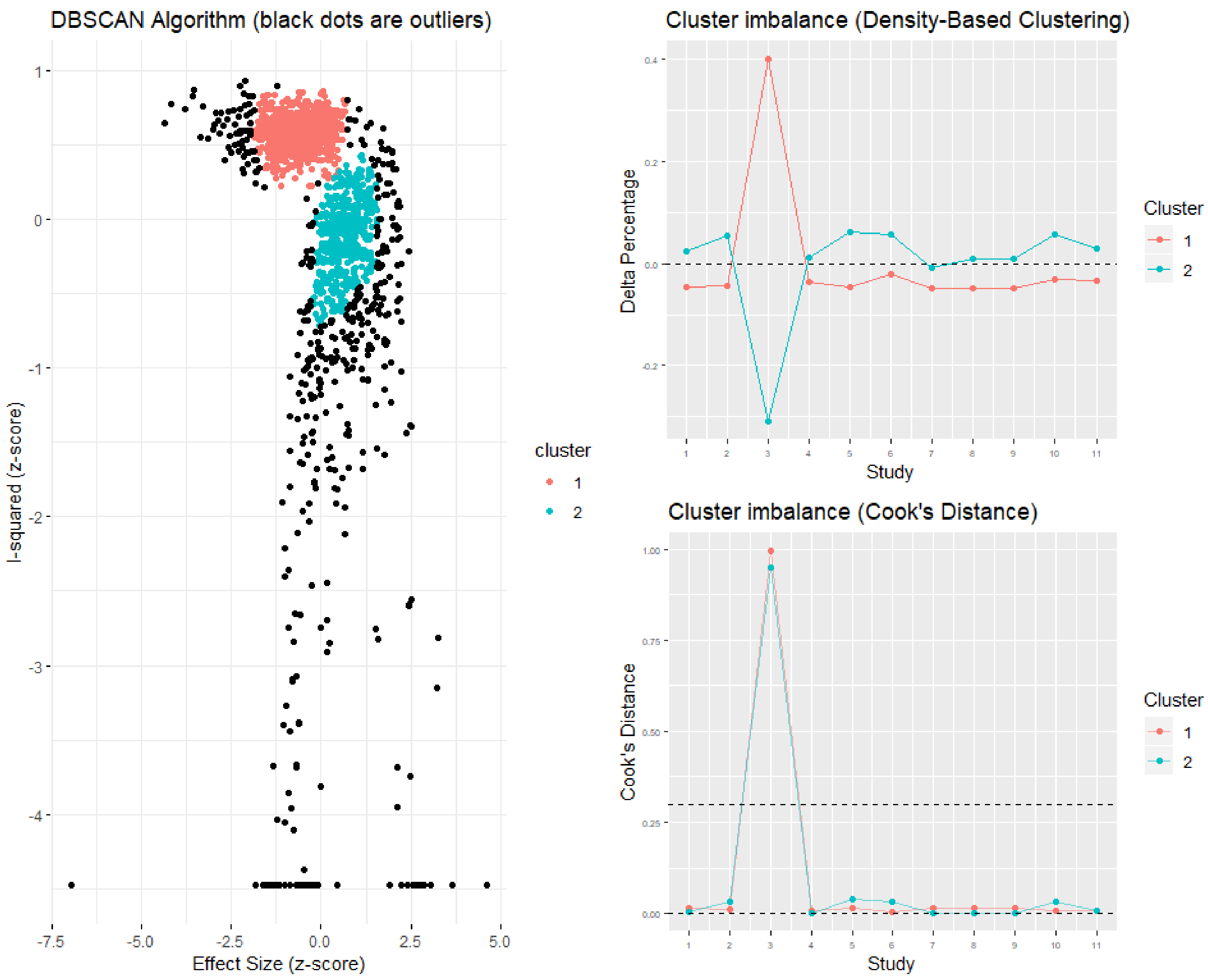

DBSCAN Algorithm (black dots are outliers)
I-squared (z-score)
Effect Size (z-score)
1
0
-1
-2
-3
-4
-7.5
-5.0
-2.5
0.0
2.5
5.0
cluster
1
2
Cluster imbalance (Density-Based Clustering)
Delta Percentage
Study
0.4
0.2
0.0
-0.2
1
2
3
4
5
6
7
8
9
10
11
Cluster
1
2
Cluster imbalance (Cook's Distance)
Cook's Distance
Study
1.00
0.75
0.50
0.25
0.00
1
2
3
4
5
6
7
8
9
10
11
Cluster
1
2

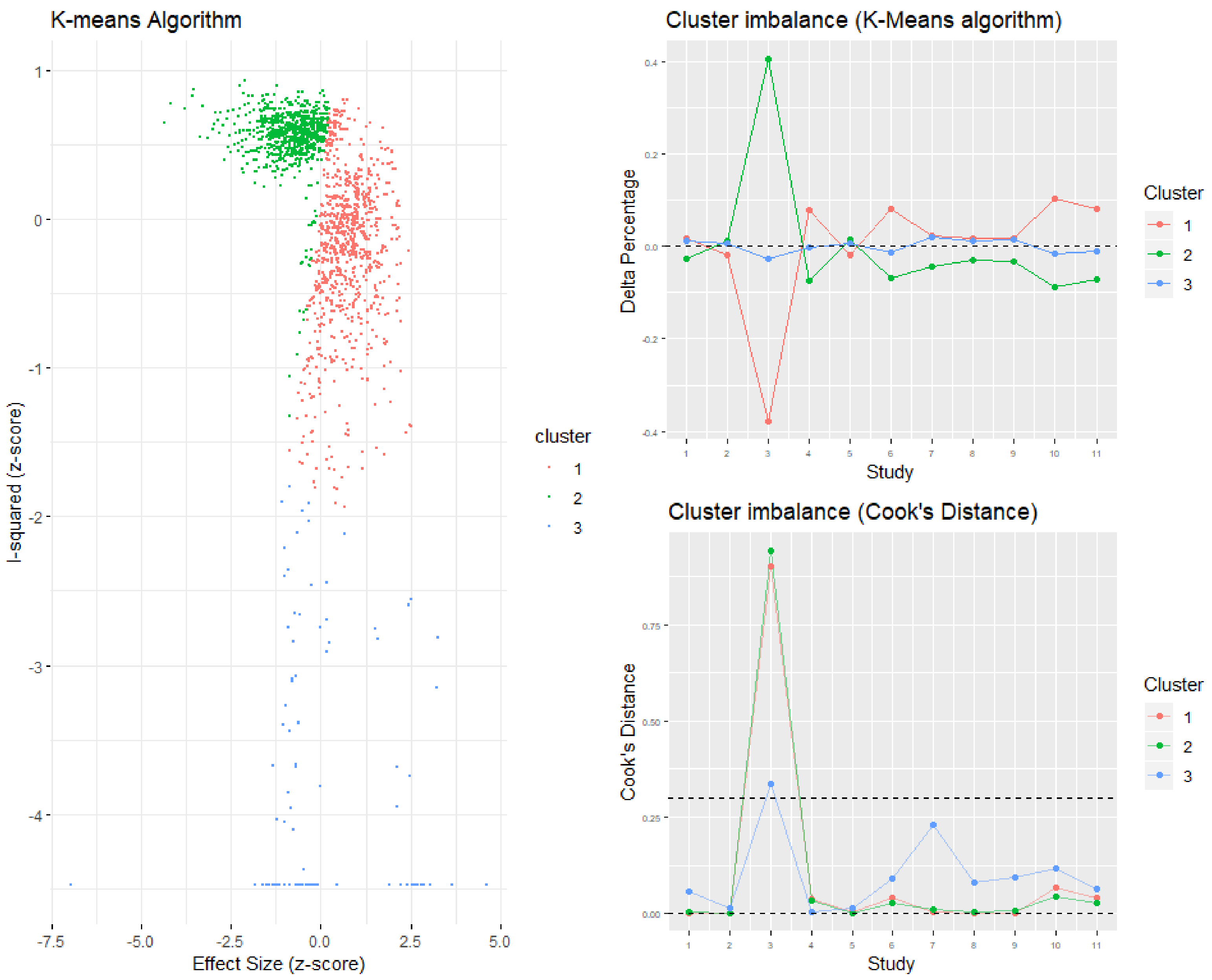

Additional Figures 1-3: To explore the patterns of the pooled effect sizes and heterogeneity we used the GOSH plot. The GOSH plot displays the pooled effect sizes of every possible subset (2^{10}–1) of the included studies in the meta-analysis against I^2. Three clustering algorithms (k-means, DBSCAN and the Gaussian Mixture Model) was used to detect clusters in the GOSH plot data and determine which of the studies contribute the most to the cluster imbalance.

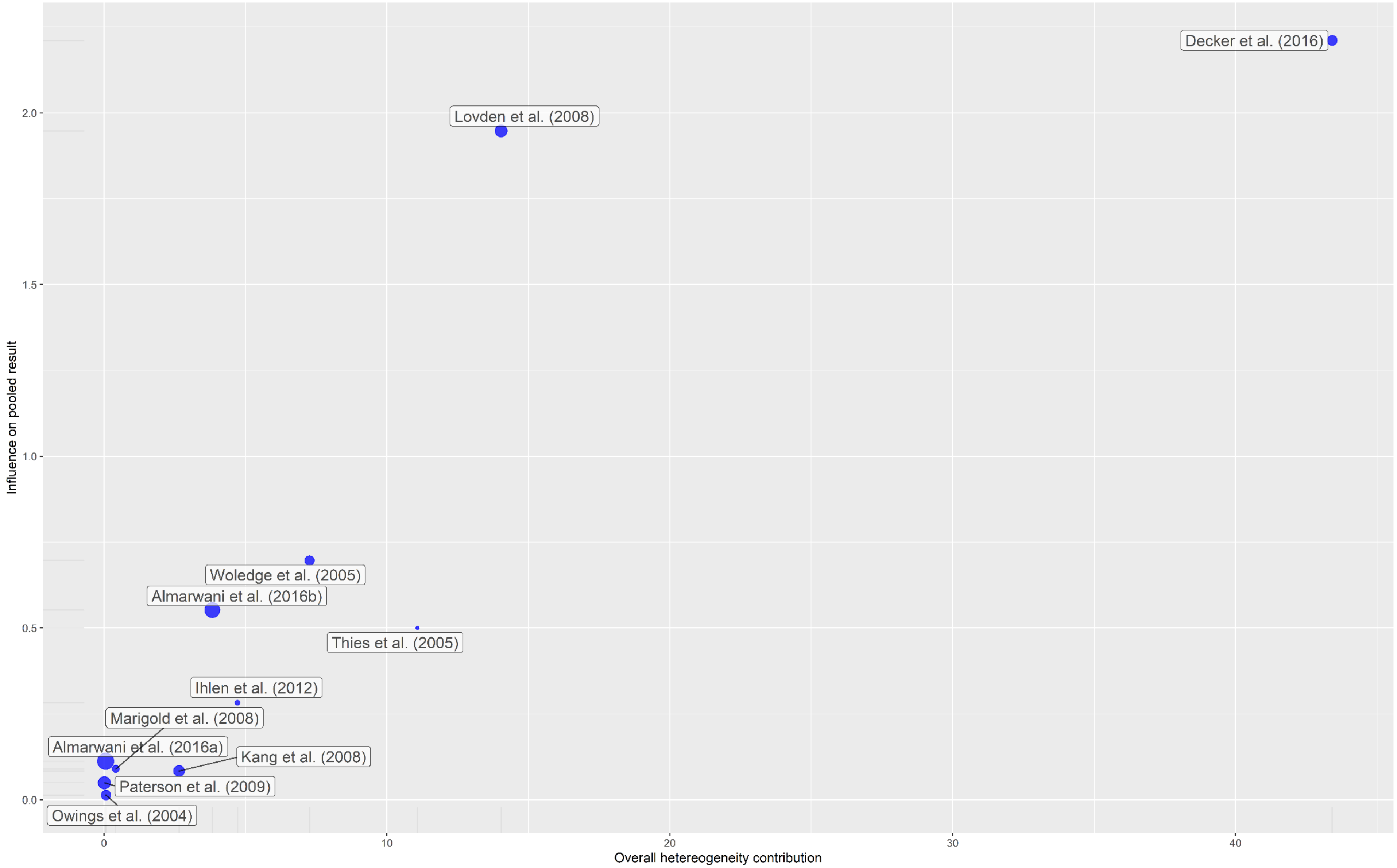

Additional Figure 4: Baujat plot showing the influence of each study on the overall Q statistic on the horizontal axis and its influence on the pooled effect size on the vertical axis

Additional Table 1. Heterogeneity sensitivity analysis. The leave-one-out-method recalculates the results of the meta-analysis by omitting one study at time.

Study	Author	ES	95% CI		I^2	rstudent	DFFITS	cook.d	cov.r	τ^2	QE.del	hat	weight	infl
1	Omitting Almarwani et al. (2016a)	0.92	0.07	1.76	0.89	-0.11	-0.06	0.01	1.43	1.07	87.47	0.10	10.23	no
2	Omitting Almarwani et al. (2016b)	0.96	0.14	1.79	0.88	-0.61	-0.22	0.06	1.30	0.96	82.83	0.10	10.10	no
3	Omitting Decker et al. (2016)	1.11	0.58	1.64	0.76	-3.80	-1.05	0.58	0.58	0.36	41.14	0.09	9.12	**yes**
4	Omitting Ihlen et al. (2012)	0.81	0.02	1.61	0.88	1.04	0.30	0.09	1.07	0.79	82.68	0.08	7.81	no
5	Omitting Kang et al. (2008)	0.97	0.15	1.79	0.89	-0.73	-0.24	0.06	1.18	0.87	84.66	0.09	9.39	no
6	Omitting Lovden et al. (2008)	0.79	-0.01	1.58	0.86	1.14	0.38	0.14	1.03	0.74	71.98	0.10	9.60	no
7	Omitting Marigold et al. (2008)	0.89	0.06	1.72	0.89	0.16	0.04	0.00	1.15	0.85	87.11	0.08	8.31	no
8	Omitting Owings et al. (2004)	0.92	0.09	1.76	0.89	-0.26	-0.09	0.01	1.19	0.88	87.47	0.09	9.04	no
9	Omitting Paterson et al. (2009)	0.91	0.07	1.75	0.89	-0.12	-0.06	0.00	1.26	0.93	87.53	0.10	9.69	no
10	Omitting Thies et al. (2005)	0.76	0.02	1.50	0.87	1.76	0.51	0.24	0.99	0.72	76.13	0.08	7.70	no
11	Omitting Woledge et al. (2005)	0.81	0.00	1.61	0.88	0.97	0.31	0.09	1.09	0.79	79.82	0.09	9.01	no

Notes: **ES** = effect size; **95% CI** = 95% confidence interval; **rstudent** = externally standardized residuals; **cook.d** = Cook's distances; **cov.r** = covariance ratios; I^2,τ^2 = leave-one-out estimates of the amount of heterogeneity; **QE.del** = leave-one-out values of the test statistics for heterogeneity; **hat** = hat values; **inf** = influential study.
